# Medida da Pressão Arterial no Braço: Há Mais do que os Olhos Enxergam

**DOI:** 10.36660/abc.20230274

**Published:** 2023-06-02

**Authors:** Rodrigo Bezerra, Audes D. M. Feitosa, Wilson Nadruz

**Affiliations:** Laboratório de Imunopatologia Keizo Asami Universidade Federal de Pernambuco Recife PE Brasil Laboratório de Imunopatologia Keizo Asami, Universidade Federal de Pernambuco, Recife, PE – Brasil; Pronto Socorro Cardiológico de Pernambuco Universidade de Pernambuco Recife PE Brasil Pronto Socorro Cardiológico de Pernambuco (PROCAPE), Universidade de Pernambuco, Recife, PE – Brasil; Departamento de Medicina Interna Faculdade de Ciências Médicas Universidade Estadual de Campinas Campinas SP Brasil Departamento de Medicina Interna, Faculdade de Ciências Médicas, Universidade Estadual de Campinas, Campinas, SP – Brasil

**Keywords:** Monitor de Pressão Arterial, Braços, Tecido Adiposo, Hipertensão, Fatores de Risco, Obesidade

A hipertensão é o fator de risco mais importante para eventos cardiovasculares na população mundial.^1 - 3^ Portanto, a identificação desse fator é fundamental para a avaliação do risco cardiovascular. A avaliação não invasiva da pressão arterial (PA) usando manguitos nos braços é o método recomendado para diagnosticar e controlar a hipertensão.^4^ No entanto, essa avaliação deve ser feita de forma adequada e precisa para evitar medidas não acuradas da PA e decisões de manejo inadequadas.

A seleção do tamanho do manguito é uma etapa fundamental na avaliação da PA e depende da circunferência do braço do indivíduo examinado. Um manguito menor do que o necessário superestima a PA, enquanto um manguito maior do que o necessário subestima a PA.^5^ Alguns estudos relataram diferenças médias na PA sistólica de 6 mmHg quando são usados manguitos de tamanho inadequado, embora discrepâncias marcadamente maiores possam ser observadas em alguns indivíduos.^6^ Nos Estados Unidos, aproximadamente 51% dos adultos com hipertensão precisam de manguitos grandes ou extragrandes, incluindo 84% daqueles com obesidade e 65% daqueles com idade entre 18 e 34 anos.^7^ Como a obesidade é altamente prevalente em todo o mundo,^8^ esses números americanos destacam a importância da avaliação sistemática da circunferência do braço para a medição precisa da PA na prática clínica.

Em artigo publicado recentemente, Souza et al.,^9^ ampliaram o conhecimento atual e forneceram dados que sugerem que a circunferência e a composição do braço podem influenciar as medidas da PA. Os autores avaliaram 489 indivíduos aparentemente saudáveis com idades entre 18 e 29 anos, que realizaram medidas de comprimento e circunferência do braço e tiveram a PA aferida simultaneamente em ambos os braços por meio de aparelhos oscilométricos com manguitos de tamanho adequado. Além disso, todos os participantes tiveram a dobra cutânea do tríceps medida e, com base nessas medidas e nos valores da circunferência do braço, foi estimado o índice de gordura do braço (IGB) de cada braço. A análise de regressão linear multivariada mostrou que a PA sistólica estava diretamente relacionada à circunferência e comprimento do braço e inversamente relacionada ao IGB. Análises posteriores usando modelos de *machine learning* (ML) confirmaram os resultados da análise de regressão linear e demonstraram que valores mais altos de PA sistólica foram encontrados em braços com menor IGB e maior comprimento do braço, mas, surpreendentemente, com menor circunferência do braço. Os autores concluíram que as medições da PA sistólica são subestimadas nos braços com maior IGB. Embora os achados sejam interessantes e provocativos, alguns aspectos do estudo merecem comentários adicionais.

Primeiro, apesar da escassez de evidências avaliando a associação entre IGB e PA, estudos anteriores demonstraram que a PA está inversamente relacionada à massa de gordura do braço^10^ e diretamente relacionada à massa muscular do braço.^11^ Esses dados reforçam a noção de que a variação na composição tecidual do braço pode influenciar a medida da PA. Como bem discutido por Souza et al.,^9^ a menor PA associada a maior IGB poderia ser devida à menor densidade de tecido adiposo que ofereceria menor resistência para a compressão do manguito da artéria braquial, gerando assim menor leitura da PA em braços com mais gordura. No entanto, para confirmar a suposição de que a PA sistólica é de fato subestimada em braços com IGB mais alto, ainda são necessários mais estudos que validem medidas indiretas de PA com medidas de PA intra-arterial em braços com uma ampla gama de IGB.

Em segundo lugar, os autores avaliaram uma amostra de jovens aparentemente saudáveis, sem hipertensão nem uso de medicamentos anti-hipertensivos. Esses indivíduos são notavelmente distintos daqueles que geralmente procuram atendimento médico para diagnóstico e tratamento da hipertensão. Resta estabelecer se a associação entre IGB e PA é reprodutível em idosos hipertensos com maior enrijecimento da artéria braquial e consequentemente maior resistência à compressão do manguito.^12^

Em terceiro lugar, os autores usaram modelos de ML como uma estratégia adicional para avaliar a relação entre as variáveis do braço e a PA. Esta abordagem tem sido cada vez mais utilizada na medicina cardiovascular e tem grande potencial para encontrar insights ocultos sem ser explicitamente programada para onde procurar.^13 , 14^ No entanto, os modelos de ML também têm limitações e seu desempenho depende de várias variáveis, incluindo qualidade de dados, métodos de validação, estratégia de integração de dados, escolha de algoritmo de ML e evidência ortogonal.^13^ Algumas dessas questões podem estar envolvidas nos achados contraditórios relatados pelos autores referentes à relação entre circunferência do braço e PA quando avaliadas por análise de regressão clássica e modelos de ML.

Apesar das considerações anteriores, o estudo de Souza et al.,^9^ merece destaque porque levanta a hipótese de que a avaliação não só da circunferência do braço, mas também da composição da gordura do braço pode ser relevante para uma avaliação precisa da PA. Novas pesquisas são necessárias para avaliar e validar o impacto do IGB na medição da PA em diferentes cenários clínicos.
