# Supplementary material for: Medida da Pressão Arterial no Braço: Há Mais do que os Olhos Enxergam
Source: Arq Bras Cardiol. 2023 Jun 2;120(5):e20230274. [Article in Portuguese] doi: 10.36660/abc.20230274 (PMC10263400; doi:10.36660/abc.20230274)
Supplement: Translation

# Translation: Blood Pressure Measurement: There’s More Than Meets the Arm

**Authors:** Rodrigo Bezerra, Audes D. M. Feitosa, Wilson Nadruz

**Affiliations:** Brazil Laboratório de Imunopatologia Keizo Asami, Universidade Federal de Pernambuco, Recife, PE – Brazil; Brazil Pronto Socorro Cardiológico de Pernambuco (PROCAPE), Universidade de Pernambuco, Recife, PE – Brazil; Brazil Departamento de Medicina Interna, Faculdade de Ciências Médicas, Universidade Estadual de Campinas, Campinas, SP – Brazil

**Keywords:** Blood Pressure Monitors, Arms, Adipose Tissue, Hypertension, Risk Factors, Obesity

Hypertension is the most important risk factor for cardiovascular events in the population worldwide.^1 - 3^ Therefore, the identification of this risk factor is crucial for the assessment of cardiovascular risk. Non-invasive evaluation of blood pressure (BP) using cuffs in the arms is the recommended method for diagnosing and managing hypertension.^4^ However, this evaluation must be done properly and accurately to avoid suboptimal BP measurement and inappropriate management decisions.

The selection of cuff size is a fundamental step in evaluating BP and depends on the arm circumference of the examined individual. A smaller-than-required cuff overestimates BP, while a larger-than-required cuff underestimates BP.^5^ Some studies have reported average differences in systolic BP of 6 mmHg when inappropriately sized cuffs are used, even though markedly greater discrepancies can be observed in some individuals.^6^ In the United States, approximately 51% of adults with hypertension need large or extra-large cuffs, including 84% of those with obesity and 65% of those aged 18-34.^7^ Because obesity is highly prevalent worldwide,^8^ these American figures highlight the importance of systematic evaluation of arm circumference for accurate BP measurement in clinical practice.

In a recently published article, Souza et al.^9^ expanded the current knowledge and provided data suggesting that arm circumference and composition may influence BP measurements. The authors evaluated 489 apparently healthy individuals aged 18-29 years who underwent arm length and circumference measurements and had BP simultaneously measured in both arms using oscillometric devices with appropriate cuff sizes. In addition, all participants had their triceps skinfold thickness measured, and based on these measurements and arm circumference values, the arm fat index (AFI) of each arm was estimated. Multivariable linear regression analysis showed that systolic BP was directly related to arm circumference and length and inversely related to AFI. Further analysis using machine learning (ML) models confirmed the results of linear regression analysis and demonstrated that higher values of systolic BP were found in arms with lower AFI and higher arm length but, surprisingly, with lower arm circumference. The authors concluded that systolic BP measurements are underestimated in arms with higher AFI. Although the findings are interesting and provocative, some study aspects deserve further comments.

First, despite a paucity of evidence evaluating the association between AFI and BP, previous reports demonstrated that BP is inversely related to arm fat mass^10^ and directly related to arm muscle mass.^11^ These data strengthen the notion that variation in arm tissue composition may influence BP measurements. As nicely discussed by Souza et al.,^9^ the lower BP associated with greater AFI could be due to the lower density of adipose tissue that would offer less resistance for cuff compression of the brachial artery, thus generating a lower BP reading in arms with more fat. However, to confirm the assumption that systolic BP is indeed underestimated in arms with higher AFI, further studies validating indirect BP measurements with intra-arterial BP measurements in arms with a wide range of AFI are still needed.

Second, the authors evaluated a sample of apparently healthy young individuals who did not have hypertension or were not using antihypertensive medications. These individuals are remarkably distinct from those who usually seek medical care for hypertension diagnosis and management. Whether the association between AFI and BP is reproducible in elderly hypertensive individuals with greater brachial arterial stiffening and consequent greater resistance for cuff compression^12^ remains to be established.

Third, the authors used ML models as an additional strategy to evaluate the relationship between arm variables and BP. This approach has been increasingly used in cardiovascular medicine and has great potential to find hidden insights without being explicitly programmed where to look.^13 , 14^ However, ML models also have limitations, and their performance depends on several variables, including data quality, validation methods, data integration strategy, choice of machine learning algorithm, and orthogonal evidence.^13^ Some of these issues might be involved in the contradictory findings of arm circumference and BP relationship when evaluated by classical regression analysis and ML models reported by the authors.

Despite the above considerations, the study by Souza et al.^9^ deserves to be highlighted because it raises the assumption that evaluation not only of arm circumference but also of arm fat composition may be relevant for an accurate assessment of BP. New research is needed to evaluate and validate the impact of AFI on BP measurement in distinct clinical scenarios.
